# microRNA miR-34a Regulates Cytodifferentiation and Targets Multi-signaling Pathways in Human Dental Papilla Cells

**DOI:** 10.1371/journal.pone.0050090

**Published:** 2012-11-30

**Authors:** Mian Wan, Bo Gao, Feifei Sun, Yin Tang, Ling Ye, Yi Fan, Ophir D. Klein, Xuedong Zhou, Liwei Zheng

**Affiliations:** 1 State Key Laboratory of Oral Diseases, Sichuan University, Chengdu, Sichuan, China; 2 West China School of Stomatology, Sichuan University, Chengdu, Sichuan, China; 3 Program in Craniofacial and Mesenchymal Biology and Departments of Orofacial Sciences and Pediatrics, University of California San Francisco, San Francisco, California, United States of America; University of Southern California, United States of America

## Abstract

Odontogenesis relies on the reciprocal signaling interactions between dental epithelium and neural crest-derived mesenchyme, which is regulated by several signaling pathways. Subtle changes in the activity of these major signaling pathways can have dramatic effects on tooth development. An important regulator of such subtle changes is the fine tuning function of microRNAs (miRNAs). However, the underlying mechanism by which miRNAs regulate tooth development remains elusive. This study determined the expression of miRNAs during cytodifferentiation in the human tooth germ and studied miR-34a as a regulator of dental papilla cell differentiation. Using microarrays, miRNA expression profiles were established at selected times during development (early bell stage or late bell stage) of the human fetal tooth germ. We identified 29 differentially expressed miRNAs from early bell stage/late bell stage comparisons. Out of 6 miRNAs selected for validation by qPCR, all transcripts were confirmed to be differentially expressed. miR-34a was selected for further investigation because it has been previously reported to regulate organogenesis. miR-34a mimics and inhibitors were transfected into human fetal dental papilla cells, mRNA levels of predicted target genes were detected by quantitative real-time PCR, and levels of putative target proteins were examined by western blotting. ALP and DSPP expression were also tested by qPCR, western blotting, and immunofluorescence. Findings from these studies suggested that miR-34a may play important roles in dental papilla cell differentiation during human tooth development by targeting NOTCH and TGF-beta signaling.

## Introduction

Odontogenesis involves three major processes: morphogenesis, histogenesis, and cytodifferentiation [1]. Cytodifferentiation results in generation of functional ameloblasts and odontoblasts, which form enamel and dentin matrix, respectively. Terminal differentiation, which is controlled by cell-matrix interactions involving several signaling pathways, starts from the bell stage. The signaling interactions between ectoderm-derived dental epithelium and neural crest-derived mesenchyme are regulated by several pathways, including TGF-beta, SHH, WNT, FGF, and NOTCH [2], [3], [4]. These growth factors interact in an intricate network regulated by spatial and temporal expression during odontogenesis [5]–[8]. Recent studies indicate that subtle changes in the activity of these major signaling pathways can have dramatic effects on tooth growth, thus demonstrating the importance of the precise control of signaling during tooth development [3], [4], [7], [9]–[14]. The regulation of tooth development by major signaling pathways has been studied [15]–[20], but the fine tuning of this network via microRNAs (miRNAs) has not yet been fully elucidated.

miRNAs are small non-coding RNAs of approximately 18–22 nucleotides (nt) that regulate gene function post-transcriptionally [21], [22]. miRNAs are transcribed from endogenous miRNA genes and generate primary (pri-) miRNAs. pri-miRNAs are processed into single hairpins or precursor miRNAs (pre-miRNAs) by the RNAase III enzyme Drosha in the nucleus. pre-miRNAs are then shuttled into the cytoplasm by Exportin-5 and further processed by the RNAase enzyme Dicer to generate mature miRNAs. miRNAs function in the form of ribonucleoproteins called miRISCs (miRNA-inducing silencing complexes) [22], which comprise Argonaute and GW-182 family proteins. miRISCs use the miRNAs as guides for the sequence-specific silencing of messenger RNAs that contain complementary sequence through inducing the degradation of the mRNAs or repressing their translation [23]–[25]. miRNAs are able to regulate the expression of multiple targets by binding to the 3′-UTR of genes. A single miRNA can target several target genes, and conversely several miRNAs can target a single gene [26]–[28]. More and more developmental and physiological processes have been found to rely on fine tuning by miRNAs [29]–[31].

To date, several studies have shown that miRNAs play a critical role in tooth development [16]–[20]. Via microarrays, miRNA expression profiles of the murine first mandibular molar tooth germ during specific developmental stages (E15.5, P0 and P5) have been established. The results indicated that the expression of miRNAs changes dynamically over time and suggested that miRNAs may be involved in the process of tooth development [17]. Following this, the function of miRNAs in tooth development was further addressed. Conditional inactivation of miRNAs in tooth epithelial cells with the *Pitx2-Cre* as early as E10.5 led to branched and multiple incisors lacking enamel and cuspless molars, indicating the overall fine-tuning roles of miRNAs [19]. However, later epithelial deletion of Dicer-1 with *K14-Cre* did not induce major tooth defects [16]. A recent study of *Wnt1-Cre*/*Dicer* ^fl/fl^ mice showed an arrest or absence of teeth development [32]. Extra incisor tooth formation was found in *Shh-Cre*/Dicer^fl/fl^ mice, whereas molars showed no significant anomalies [32].

microRNA miR-34a was previously reported to be involved in the proliferation and apoptosis of stem cells [33], [34] and as a suppressor of tumorigenesis [26], [34]. The miR-34a responsive genes are highly enriched for those that regulate cell cycle progression, cellular proliferation, apoptosis, DNA repair, and angiogenesis, thereby providing a functional basis for the regulatory role of this miRNA in organogenesis. However, the role of miR-34a in tooth development is still unknown.

The present study aimed to investigate the expression of miRNAs during cytodifferentiation in the developing human tooth and to study miR-34a as a regulator of dental papilla cell differentiation.

## Materials and Methods

### Ethics statement

All human tissues were collected from legally aborted fetuses at West China Women and Children's Hospital under approved guidelines set by Sichuan University. Written informed consent of all human subjects who participated in the experimental investigation was obtained. The study and the consent procedure were approved by Ethical Committees of West China School of Stomatology, Sichuan University and State Key Laboratory of Oral Diseases.

### Sample collection

Human tooth buds of either early bell stage (16 wks) or late bell stage (20 wks) were obtained from fetal cadaver tissue within 3 hours after legal abortion, under the guidelines of the West China School of Stomatology, Sichuan University Committee on Human Research. Tissues were placed on ice in penicillin/streptomycin in PBS, and teeth were dissected from the mandibles under a laminar flow hood. Tooth organs were preserved in RNA*later*® [35] (Life Technology Corporation, Foster city, CA, USA) at −80°C before RNA extraction. Total RNA including small RNAs were purified using the miRNeasy Mini Kit (Qiagen Inc, Valencia, CA, USA) according to the manufacturer's instructions. Agilent 2100 bioanalyzer was used to determine samples quality.

### Microarray hybridization and data analysis

In this study, early bell stage tooth buds and late bell stage tooth buds with three biological repeats for each were tested. RNA quality and concentration were determined using a 2100 Bioanalyzer (Agilent Technologies Inc, Wilmington, DE, USA). Agilent microarray hybridization was carried out by the ShanghaiBio Corporation. miRNA microarray profiling was performed as previously described [36]. Data analysis was performed by using GeneSpring GX software (Agilent). A miRNA was designated as highly expressed if expression in late bell stage was >1.5-fold compared to that in early bell stage.

### Validation of microarray results using real-time RT-PCR

The purified total RNA including small RNAs were used as templates. Reverse-transcription was performed with the TaqMan® MicroRNA Reverse Transcription Kit using small RNA-specific RT primer. The reactions were incubated at 16°C for 30 min, 42°C for 30 min and 85°C for 5 min, chilled on ice for 5 min, and the cDNA was stored at −20°C. The qRT-PCR was performed with the TaqMan® Small RNA Assay following the manufacturer's instructions in 20 µl reaction mixtures. U6 was used as endogenous control to normalize Ct values obtained for each gene. miRNA expression was compared by ΔΔCt [37]. Data were compared by one-way ANOVA followed by the post-hoc Tukey's test.

### Cell culture

Human fetal dental papilla cells were cultured as described below. Human tooth buds were obtained from fetal cadaver tissue within 3 hours after legal abortion, under the guidelines of the West China School of Stomatology, Sichuan University Committee on Human Research. Tissues were placed on ice in penicillin/streptomycin in PBS, and tooth buds were dissected from mandibles under a laminar flow hood. Tissues were dispersed by the addition of 2 mg/ml Collagenase/Dispase at 37°C for 1.5 hr. Reaction was quenched and cells were plated by DMEM medium supplemented with 15% FBS, 1% penicillin/streptomycin. After 1 day of culture, cells were attached to the plate.

### Oligonucleotide transfection

miR-34a mimics and miR-34a inhibitors (anti–miR-34a, chemically modified antisense oligonucleotides designed to specifically target mature miR-34a) were synthesized by Ribobio, Guangzhou, China. Oligonucleotide transfection was performed with Lipofectamine 2000 reagents (Life Technology). The final concentration of miR-34a mimics or miR-34a inhibitors in the transfection system was 50 nM and 200 nM, respectively. Oligonucleotide was transfected at 40% confluency.

### RNA extraction and quantitative real time RT-PCR (qRT-PCR)

Cells were harvested after treatment. Total RNA including small RNAs was isolated with miRNeasy Mini Kit (Qiagen). RNA concentration was determined by NanoDrop ND-1000 (Thermo Fisher Scientific, Inc, Wilmington, DE, USA). miR-34a Reverse-transcription was performed with the TaqMan® microRNA Reverse Transcription Kit using miR-34a specific RT primer. The reactions were incubated at 16°C for 30 min, 42°C for 30 min and 85°C for 5 min, chilled on ice for 5 min, and the cDNA was stored at −20°C. cDNA synthesis for messenger RNA was performed using the SuperScriptIII First-Strand Synthesis System (Life Technology). The qRT-PCR for miR-34a was performed with the TaqMan® Small RNA Assay following the manufacturer's instructions in 20 µl reaction mixtures. U6 were used as endogenous control to normalize Ct values obtained for each gene. miRNA expression was compared by ΔΔCt [36]. Data were compared by one-way ANOVA followed by the post-hoc Tukey's test. The mRNA level of miR-34a target genes (*NOTCH1*, *FGF2*, *BMP7*, *LEF1*, *GLI2*) and differentiation markers *DSPP* and *ALP* were examined by quantitative real-time PCR using an ABI 7900 system (Applied Biosystems, Foster City, CA, USA). Primers and probes sets, including an endogenous *GAPDH* control, were purchased from Applied Biosystems. mRNA expression was compared by ΔΔCt. Data were compared by one-way ANOVA followed by the post-hoc Tukey's test.

### Western blotting

Total cellular protein was extracted using the Reagent kit (KeyGEN, Nanjing, Jiangsu, China) after mimics or inhibitors treatment. Protein concentration was determined using the BCA protein assay reagent (Beyotime, Haimen, Jiangsu, China). An equal amount of each sample (30 µg) was electrophoresed on either 6% SDS-PAGE or 12% SDS-PAGE and transferred to Nitrocellulose membrane. After blocking with non-fat dried milk, membranes were probed with primary antibody: mouse anti-GAPDH (D-6)(1∶200), mouse anti-DSPP (LFMb-21)(1∶200), rabbit anti-FGF-2 (H-131)(1∶200), mouse anti-GLI-2 (1∶200) (Santa Cruz Biotechnology, Santa Cruz, CA, USA), rabbit anti-NOTCH-1 (1∶500), rabbit anti-LEF1 (EP2030Y)(1∶5000), rabbit anti-BMP7 (1∶500) or rabbit anti-Alkaline Phosphatase, tissue non-specific (1∶200) (Abcam Inc., Cambridge, MA, USA). Blots were then incubated with goat anti-rabbit IgG-HRP or goat anti-mouse IgG-HRP (Santa Cruz Biotechnology) and detected with a chemiluminescent reagent kit (Millipore, Billerica, MA, USA). GAPDH expression served as an internal control. For image analysis, the film was scanned with Imaging Densitometer GS-700 (Bio-Rad, Benicia, CA, USA) and analyzed.

### Immunofluorescence

Human fetal dental papilla cells were fixed with 4% Paraformaldehyde in a chamber slide. Then the cells were incubated with 3% goat serum, 0.1% BSA and 0.1% Triton (blocking solution) for 1 hr at RT. Primary antibody mouse anti-DSPP (LFMb-21)(1∶ 100), rabbit anti-FGF-2 (H-131)(1∶100), mouse anti-GLI-2 (1∶100)(Santa Cruz Biotechnology), rabbit anti-NOTCH-1 (1∶100), rabbit anti-LEF1 (EP2030Y)(1∶5000), rabbit anti-BMP7 (1∶100) or rabbit anti-Alkaline Phosphatase, tissue non-specific (1∶100)(Abcam) was incubated with cells overnight at 4°C. After thoroughly washing, the slides were incubated with Fluorescein-Conjugated AffiniPure Goat Anti-Mouse IgG (H+L)(1∶100) and Rhodamine (TRITC)-Conjugated AffiniPure Goat Anti-Rabbit IgG (H+L)(1∶100) for 1 hr. Nuclei were counterstained with 0.5 g/mL Hoechst 33342 (Life Technology) in the dark for 5 min. After mounting, the slides were photographed with a Nikon Eclipse 300 fluorescence microscope (Compix Inc, Sewickley, PA, USA).

### Statistical analysis

All experiments were performed independently at least three times in triplicate. Bands from western blotting were quantified with Image Lab software (Bio-Rad, Hercules, CA). Relative protein and mRNA levels were calculated in comparison to internal GAPDH standards. Relative miRNA levels were calculated in comparison to internal U6 standards. Numerical data are presented as mean±SD. The difference between means was analyzed with one-way ANOVA. Differences were considered significant when P<0.05. All statistical analyses were done with the software SPSS13.0 (SPSS Inc. Chicago, USA).

## Results

### Identification of differentially expressed miRNAs

**Figure 1:**
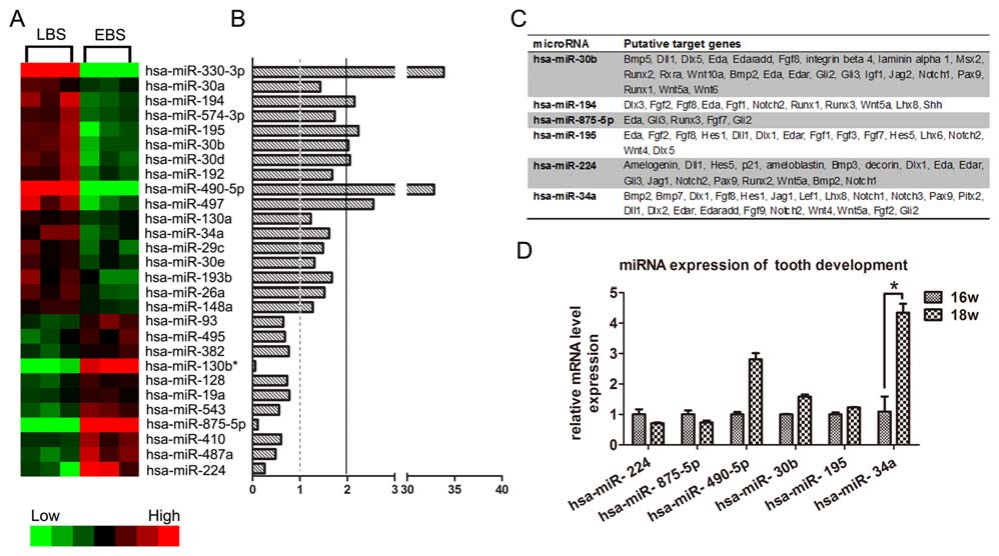
Differentially expressed miRNAs between early bell stage and late bell stage of human tooth germ. (A) Heat-map of miRNAs that are differentially expressed at least 1.5-fold (p<0.05) between early bell stage and late bell stage. (LBS: late bell stage; EBS: early bell stage) (B) Bar graph showing fold changes. (C) Predicted target genes of differentially expressed miRNAs in all three data bases (miRBase Target 5.0, the TargetScanHuman5.1, and the miRNAMap 2.0. (D) Validation of microarray data by qPCR.

The developing human tooth germ can be divided into 4 main stages: dental lamina, bud stage, cap stage and bell stage. During the bell stage, cytodifferentiation starts and generates extracellular cell matrix. Although it is well described that specific molecular pathways are involved in tooth development, little is known regarding the role of miRNAs in this process. Therefore, we set out to identify miRNAs that could be involved in the differentiation of dental papilla cells. We isolated total RNA including microRNAs from early and late bell stages of the human tooth germ and miRNA microarray analysis was performed. From a total of 1887 human miRNAs assayed, we identified transcripts that were differentially expressed with adjusted *p*-values of less than 0.05 between early bell stage and late bell stage human tooth germ. Heat maps and fold differences of the differentially expressed miRNAs in either early bell stage or late bell stage (Fig. 1A, B) comparisons were produced.

### Data analysis of microarray

For each differentially expressed, array-identified miRNA, the predicted targets were retrieved from the tooth-oriented miRNA target prediction database, miRTooth, derived from the Bite-it database (http://bite-it.helsinki.fi), which has been created including miRBase Target 5.0, the TargetScanHuman5.1, and the miRNAMap 2.0 [38]. Only the predicted targets expressed in dental papilla and found in all 3 prediction databases were retained (Fig. 1C).

### miR-34a expression is significantly upregulated in late bell stage of human tooth germ

Microarray results suggested that miR-34a expression increased from early bell stage to late bell stage. Microarray results were validated by using real-time RT-PCR and showed that miR-34a was significantly upregulated in late bell stage tooth germs (Fig. 1D).

### miR-34a regulates the differentiation of human dental papilla by downregulating ALP and upregulating DSPP

**Figure 2:**
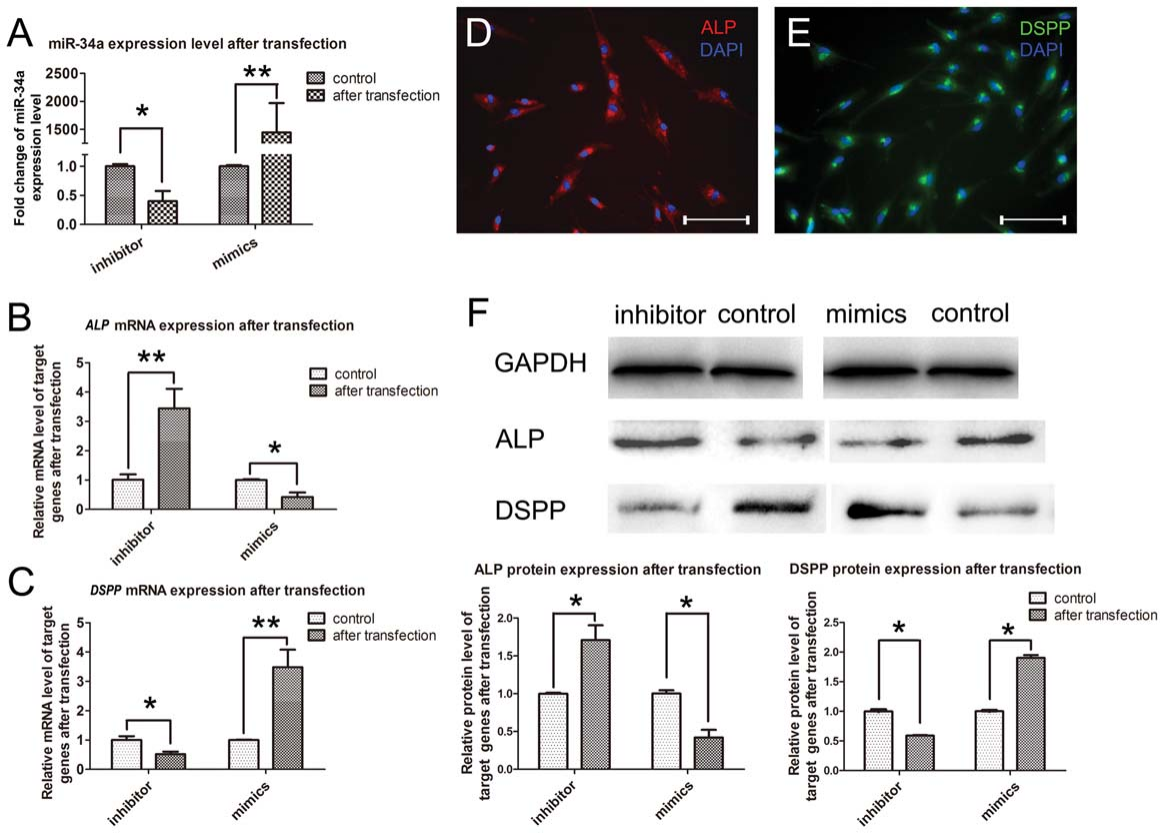
miR-34a regulated cytodifferentiation of human dental papilla cells *in vitro*. (A) miR-34a expression was downregulated after miR-34a inhibitor transfection, and was upregulated after miR-34a mimic transfection. (B) mRNA level of *ALP* was significantly upregulated after miR-34a inhibitor transfection, and was significantly downregulated after miR-34a mimic transfection. (C) mRNA level of *DSPP* was significantly downregulated after miR-34a inhibitor transfection, and was significantly upregulated after miR-34a mimic transfection. (D, E) Both protein signals of ALP and DSPP were detected by immunofluorescence in human dental papilla cells *in vitro*. (F) Western blotting results showed that protein level of both DSPP and ALP was accordingly shifted after either miR-34a mimic or inhibitor transfection. (*: *p*<0.05 as determined by one-way ANOVA test followed by the post-hoc Tukey's test. **: *p*<0.01 as determined by one-way ANOVA test followed by the post-hoc Tukey's test. Scale bar: 20 µm).

Studies have reported that miR-34a acted as a tumor suppressor in uveal melanoma cell proliferation and migration through the down-regulation of c-Met [39]. However, the function of miR-34a in cytodifferentiation during tooth development remains unknown. To further investigate the role of miR-34a in odontogenesis, we transfected human fetal dental papilla cells and detected the mRNA level of *ALP* and *DSPP* by qPCR and the protein expression level of ALP and DSPP by western blotting. miR-34a level was down-regulated after inhibitor transfection and was up-regulated after mimics transfection (Fig. 2A).

In cultured human dental papilla cells, when transfected with miR-34a mimics, the expression level of *DSPP* was upregulated, while the expression level of *ALP* was down-regulated. In contrast, when transfected with miR-34a inhibitors, the expression level of *DSPP* was down-regulated while the expression level of *ALP* was up-regulated (Fig. 2B,C).

The results from western blotting demonstrated that ALP protein level decreased in the cells transfected with miR-34a mimic, while they increased in the cells transfected with miR-34a inhibitors. In contrast, examination of DSPP protein presented the opposite results (Fig. 2F).

### miR-34a regulates cytodifferentiation of human fetal dental papilla cells by targeting *NOTCH1* and *BMP7*

**Figure 3:**
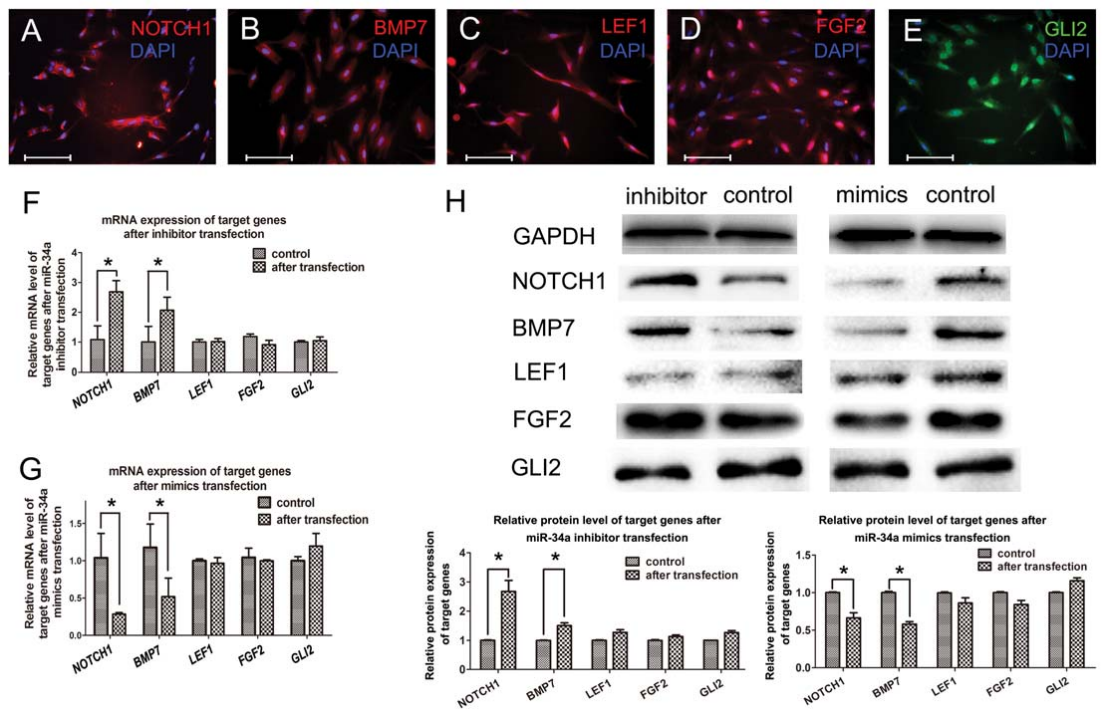
miR-34a targets signaling pathways in human dental papilla cells. (A–E) Protein signals of predicted targets of miR-34a were detected by immunofluorescence in human dental papilla cells. (F) mRNA level of *NOTCH1* and *BMP7* were upregulated by inhibitor transfection. *LEF1*, *FGF2* and *GLI2* was not affected. (G) In contrast, mRNA level of *NOTCH1* and *BMP7* were downregulated by mimic transfection. *LEF1*, *FGF2* and *GLI2* was not affected. (H) Western blotting results indicated that protein level of these targets were regulated by either miR-34a mimic or inhibitor transfection. (*: *p*<0.05 as determined by one-way ANOVA test followed by the post-hoc Tukey's test. Scale bar: 20 µm).

*LEF1*, *NOTCH1*, *FGF2*, *BMP7* and *GLI2*, which are involved in tooth development, are putative target genes of miR-34 [38]. qPCR results showed that *NOTCH1* and *BMP7* mRNA expression were down-regulated 72 hrs after miR-34a mimics transfection, while up-regulated 72 hrs after miR-34a inhibitor transfection. However, the expression level of *LEF1*, *FGF2* and *GLI2* mRNA remained the same (Fig. 3F, G).

Immunofluorescence confirmed that predicted target gene-encoded proteins, BMP7, NOTCH1, FGF2, LEF1 and GLI2, were expressed in fetal dental papilla cells (Fig. 3A–E).

Analysis of protein expression level was consistent with qPCR results. Western blotting showed that NOTCH1 and BMP7 protein level were down-regulated 72 hrs after miR-34a mimics transfection, while these were up-regulated 72 hrs after miR-34a inhibitor transfection. Interestingly, the fold changes of *NOTCH1* mRNA and NOTCH1 protein were not consistent in the same level (Fig. 3F, H). However, LEF1, FGF2 and GLI2 did not change after transfection (Fig. 3H).

## Discussion

miRNAs are important regulators of signaling pathways during morphogenesis and organogenesis, including tooth development [19]. The importance of Dicer and miRNAs during tooth development has been demonstrated, but there has been relatively little progress in the identification and characterization of the roles of specific miRNAs in tooth development.

The miRNA microarray analysis from early and late bell stage of human tooth germs yielded numerous differentially expressed miRNA transcripts. Among the differentially expressed miRNAs, miR-34a was chosen for validation by qPCR and was indeed differentially expressed in the early/late bell stages. Interestingly, we found that many target genes were transcription factors that participated in organ development and were associated with most of the tooth development signaling pathways.

miRNAs function as fine regulators during biological events by posttranscriptional mechanisms [24]. In this study, when cells were transfected with specific miRNA oligonucleotides, both the mRNA levels and the protein levels of predicted target genes were shifted. Interestingly, the fold changes of some target genes were not concordant at mRNA (Fig. 3F *NOTCH1*) and protein levels (Fig. 3H NOTCH1). This may be because miRNAs either promote degradation of target mRNA or inhibit translation posttranscriptionally.

**Figure 4:**
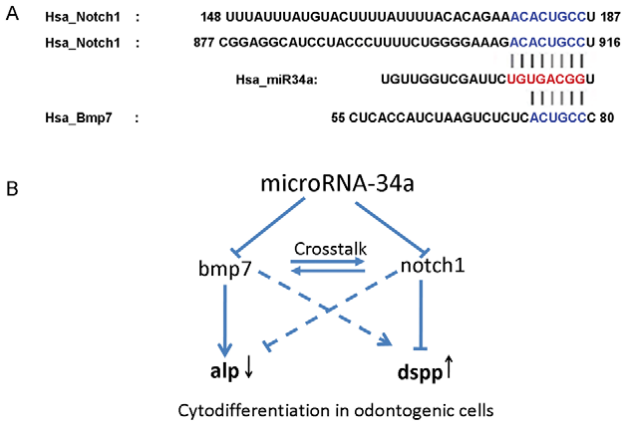
microRNA-34a regulates cytodifferentiation of human dental papilla cells via NOTCH and TGF-beta signaling pathways. (A): Putative binding sites of miR-34a in *NOTCH1* and *BMP7* 3′UTRs are shown with color letters. (B) Schematic of miR-34a regulation of human dental papilla cell differentiation: miR-34a targets both *NOTCH1* and *BMP7*. By targeting *NOTCH1*, miR-34a suppresses NOTCH signals and promotes expression of DSPP in dental papilla cells. In contrast, by targeting *BMP7*, miR-34a suppresses TGF-beta signals and inhibits expression of ALP. The overall results of miR-34a regulation in human tooth development are up-regulation of odontogenic differentiation marker, DSPP, and to the contrary, down-regulation of the osteogenic maker, ALP.

Target prediction tools revealed that *LEF1* (WNT pathway), *FGF2* (FGF pathway), *BMP7* (TGF-beta pathway), *NOTCH 1* (NOTCH pathway) and *GLI2* (SHH pathway) were predicted target genes of miR-34a, and all of them are members of crucial signaling pathways involved in tooth development. Our findings revealed that at both the mRNA level and protein level both BMP7 and NOTCH1 in cells were affected after oligonucleotide transfection. This result supported the prediction that these genes can be recognized at their 3′UTRs and targeted by miR-34a. It has been well documented that activation of NOTCH signaling can inhibit cell differentiation, while suppression of the pathway leads to cell differentiation [40]–[42]. We propose (Fig. 4) that by targeting *NOTCH1*, miR-34a suppresses the NOTCH signals in developing human dental mesenchyme, resulting in cell differentiation and up-regulation of DSPP in human dental papilla cells. Meanwhile, miR-34a can target *BMP7*, which is a member of the TGF-beta signaling pathway. Involvement of BMP7 in the developmental events was suggested as early as 1995 that *BMP7 deficient mice* resulted in skeletal defects restricted to the rib cage, the skull and the hindlimbs [43] Other investigations indicated that *BMP7* was expressed in dental papilla cells which will further differentiate into odontoblasts [44]. Tooth phenotypes were exhibited in *Bmp7*-conditional knockout mice generated by Zouvelou. Conditionally knockout of *Bmp7* in dental mesenchyme resulted in missing maxillary incisors and deformed/hypoplastic mandibular incisors [44], [45]. In the present study, by suppressing the expression of BMP7, miR-34a down-regulates the expression of ALP. Previous study pointed out that *ALP* in odontoblasts decreased at late odontogenic developmental stage.[46] NOTCH signaling and TGF-beta signaling pathway are well-known to have crosstalk with each other in organogenesis through a spatial and temporal pattern [47]–[49]. In our model, the overall effect is that miR-34a regulates the cytodifferentiation of human dental papilla cells by down-regulating ALP and up-regulating DSPP.

Since there were no changes in either mRNA level and protein level of miR-34a predicted target genes *LEF1*, *FGF2* and *GLI2*, our data indicate that miR-34a does not indiscriminately affect all the pathways involved in tooth development. This further supports our hypothesis that miR-34a specifically targets NOTCH and TGF-beta signaling pathways and regulates dental cell differentiation in developing tooth germ.

In conclusion, our analyses utilizing microarray technology, qPCR, western blotting, immunofluorescence, and target prediction tools have indicated that miRNA-34a may play an important role in dental papilla cells differentiation during human tooth development by targeting several signaling pathway.
